# Effect of oral motor facilitation technique on oral motor and feeding skills in children with cerebral palsy : a case study

**DOI:** 10.1186/s12887-022-03674-8

**Published:** 2022-11-03

**Authors:** Kyoung-chul Min, Sang-min Seo, Hee-soon Woo

**Affiliations:** 1 Department of Occupational Therapy, Seoul Metropolitan Children’s Hospital, Seocho-gu, Seoul Republic of Korea; 2 Department of Occupational Therapy, Semyung University, Seyoungro 65, Jecheon city, Chungchungbuk-do Republic of Korea; 3 Department of Occupational Therapy, College of Medicine, Wonkwang University, 460 Iksandaero, Iksan city, Jeollabuk-do Republic of Korea

**Keywords:** Cerebral palsy, Oral motor facilitation technique, Oral motor function, Oral motor exercise, Oral motor therapy

## Abstract

**Background:**

Deficiencies in oral motor function and feeding skills are common in children with cerebral palsy (CP). Oral motor therapy is a useful method to improve oral motor function and feeding skills. Oral motor facilitation technique (OMFT) is a newly designed comprehensive oral motor therapy, including postural control, sensory adaptation, breathing control, sensorimotor facilitation, and direct feeding.

**Methods:**

This study was performed to identify the effect of OMFT on oral motor function and feeding skills in children with CP. A total of 21 children with CP (3–10 years, GMFCS III–V) participated in 16 weeks (16 sessions) of OMFT. The effects on oral motor function and feeding skills were assessed using the Oral Motor Assessment Scale (OMAS) before the treatment, 8 and 16 weeks after OMFT. Data were analyzed using the Friedman test and post-hoc analysis.

**Results:**

Significant improvement was found in oral motor function and feeding skills including mouth closure, lip closure on the utensil, lip closure during deglutition, control of the food during swallowing, mastication, straw suction, and control of liquid during deglutition after OMFT. Mouth closure was the most effective and mastication was the least effective item. Sixteen weeks is more effective than 8 weeks of OMFT.

**Conclusion:**

OMFT could be an effective and useful oral motor therapy protocol to improve oral motor function and feeding skills in children with CP.

## Introduction

Cerebral palsy (CP) is a neurologic disorder with sensory, cognitive, motor, and movement problems caused by non-progressive or immature infant brain lesions [1]. Feeding problem is very common in CP and the oral motor problem is one of the major causes [2, 3]. The prevalence of oral motor problems in CP is 68–90% [4, 5]. Symptoms of oral motor problems in CP include problems in efficient and safe suck-swallow-breathe (SSB) control, poor oral motor function, and decreased bolus and drooling control [5–11].

In spastic type, increased muscle tone, and poor postural control are common symptoms. However, difficulties of movement coordination and timing are major problems in dyskinetic type [2, 7]. In infant, poor SSB coordination, decreased feeding development, and lack of mouth feeding experience are major problems. As grows older, difficulties in efficient and safe feeding and eating proper food for their ages, poor chewing skills and problems in using feeding device like spoon and straw might appear [2, 7, 8].

Oral motor function is comprehensive including postural control, oral sensorimotor function, motor control, and motor learning [1, 7, 8, 11, 12]. Oral motor function is an essential part of feeding, eating, swallowing, and communication [13, 14]. Oral motor function affects feeding skills, nutritional status, and Quality Of Life (QOL) [15]. Therefore, oral motor therapy in CP is essential to improve oral motor function and feeding skills [15–19].

Oral motor therapy is an effective traditional feeding therapy [16, 18]. Oral motor therapy consists of direct manual stroking, passive sensory stimulation, and active oral motor exercise [16, 18]. For several decades, many studies verified the effects of oral motor therapy on oral motor function, swallowing, and feeding function of CP [10, 15, 17]. However, oral motor therapy in previous studies had several limitations, such as providing only simple sensory stimulation, focusing on specific oral structures, and providing oral motor therapy to CP with moderate feeding problems [10, 15, 17, 20].

To address these limitations, Min et al. [18, 21] designed the oral motor facilitation technique (OMFT). OMFT is a systematic oral motor therapy protocol to facilitate oral motor function, planning, and oral praxis through postural control, sensorimotor facilitation, voluntary participation, motor control, and motor learning [18, 21]. OMFT consists of 3 techniques including warm-up technique, key point technique, and application technique. OMFT includes 10 categories and 50 techniques [21]. Warm-up technique consists of 2 categories and 12 techniques, including sensory and treatment adaptation, postural control, and breathing control. Key point technique is composed of 7 categories and 30 techniques, such as direct manual stroking on oral structure for lip closure, tongue movement, and chewing function. Application technique consists of 1 category and 8 techniques, including direct food control and supporting chewing and swallowing. OMFT comprises nine basic concepts; (1) oral adaptation, (2) oral awareness, (3) sensory stimulation, (4) proprioceptive activation, (5) breathing control, (6) neural facilitation, (7) structural elongation, (8) muscular strengthening, and (9) voluntary exercise [18]. OMFT is suitable for patients who cannot participate actively because of a lack of consciousness as well as infants and toddlers. OMFT can be customized by individual functions, from basic sensory stimulation to real food processes.

Therefore, the aim of this study was to investigate the effectiveness of OMFT on oral motor function and feeding skills in CP using a standardized oral motor assessment tool.

## Materials and methods

### Participants

27 parents of children applied after seeing the guide on the research purpose and process in Seoul Metropolitan Children’s Hospital. 6 children with seizures or tube feeding were excluded according to exclusion criteria. A total of 21 children with CP (15 boys, 6 girls) with feeding problems, 3–10 years participated. Inclusion criteria were as follows: scores below 10 in baseline OMAS, participation in more than 80% of the whole process, no experience of OMFT, children with head and neck control problems, and audible, visible perceptual deficiency. Exclusion criteria were children with seizures, oral structure problems, tube feeding, and aspiration. Gross Motor Function Classification Scale(GMFCS) level and type of CP was identified from the medical records. All participants recruited from one children’s rehabilitation hospital in Seoul. Written consents were collected from all parents of participants.

### Procedures

This study was an interventional and case study. Participants received 16 sessions of OMFT for 16 weeks. The Oral Motor Assessment Scale (OMAS) was assessed before the OMFT and 8 and 16 weeks after OMFT. The first author, an occupational therapist who participated in developing the OMFT, carried out all the processes for the consistency of the study. In previous studies, the period of therapy was during 8–24 weeks [10, 15, 22], and at least 12 sessions of oral motor therapy is effective [15]. Therefore, the total OMFT periods were set to 16 sessions in 16 weeks in this study.

### Outcome measures

OMAS is a standardized oral motor function assessment tool for assessing oral motor problems in CP [23]. It assesses seven oral motor functions and feeding skills by direct observation of children’s mealtime without additional participation and following directions. Items of OMAS are mouth closure, lip closure on the utensil, lip closure during deglutition, control of the food during swallowing, mastication, straw suction, and control of liquids during deglutition. Each item takes 30 s. The rating system was 0 = passive, 1 = subfunctional, 2 = semi-functional, and 3 = functional. Higher score indicates high oral motor function and feeding skills. Inter-rater reliability was Kappa > 0.85, and intra-rater reliability was Kappa > 0.90. In this study, the assessment was performed in a quiet feeding therapy room. The participants sat in a feeder seat to support the trunk and head. The researcher asked the caregivers (all the children’s mothers) to feed their child in the following order: soft, hard and liquid food in the same as usual mealtime.

### Treatment protocol

OMFT was provided in the order of warming up technique, key point technique and application technique, with the researcher’s direct manual stroking. Details of each technique are as follows: (1) warming up technique: postural control of the face and neck, nasal breathing facilitation, sensory adaptation, and awareness; (2) key point technique: oral structure facilitation and chewing; and (3) application technique: real food control (Table 1). The treatment was performed in a quiet feeding therapy room. The participants sat in a feeder seat to support the trunk and head. Sixteen sessions of OMFT was provided individually, depending on the oral function and development level.

**Table 1:** Oral motor facilitation technique

Techniques	Classification	Contents
Warming up	2 categories 12 techniques	Postural control, Neck facilitation Sensory adaptation, Breathing control Oral preparation
key point	7 categories 30 techniques	Direct facilitation on oral structures(face, cheek, gum, tongue and jaw) Chewing
Application	1 category 8 techniques	Direct approach for acceptation and swallowing food Real food control

### Statistical analysis

Differences among periods of OMFT were analyzed using the Friedman test. The significance level (α) was set to 0.05. If the difference was significant, the effect of different periods of OMFT was identified by the Wilcoxon signed-rank test. To reduce type I error, the significance level (α) was modified to 0.017 (0.05/3) by Bonferroni correction. Window SPSS ver. 25 was used for analyzing all the results.

## Results

### Study participants

Participants were 15 boys (71.4%) and 6 girls (28.6%). The average age was 5.88 (SD = 1.98). GMFCS level was III–V (III, 14.3%; IV, 33.3%; V, 52.4%). Most of the subjects were spastic quadriplegia (16/21; 76.2%)(Table 2). Most of the children with GMFCS Level IV-V couldn’t control their trunk and neck, move independently and follow instruction. Seventeen children participated all sessions, the others missed 1–2 sessions. Almost participants adapted and accepted OMFT in 4 session.

**Table 2:** Demographic characteristics of the participants

Variables		*n*	%
Gender	Male	15	71.4
	Female	6	28.6
Gestational age (weeks)	Average (M ± SD)	35.24 ± 3.52	
	Range	30–40	
Age at assessment (years)	3–4	10	48.7
	5–6	6	28.6
	7–8	3	14.3
	9–10	2	9.6
	Average (M ± SD)	5.88 ± 1.98	
	Range	3.2–10.3	
GMFCS	Level III	3	14.3
	Level IV	7	33.3
	Level V	11	52.4
Primary motor type	Spastic	16	76.2
	Dyskinetic	4	19.1
	Mixed	1	4.8
Motor distribution	Quadriplegia	16	76.2
	Diplegia	5	23.8

GMFCS: Gross Motor Function Classification System

### Effect on oral motor function

**Fig. 1:**
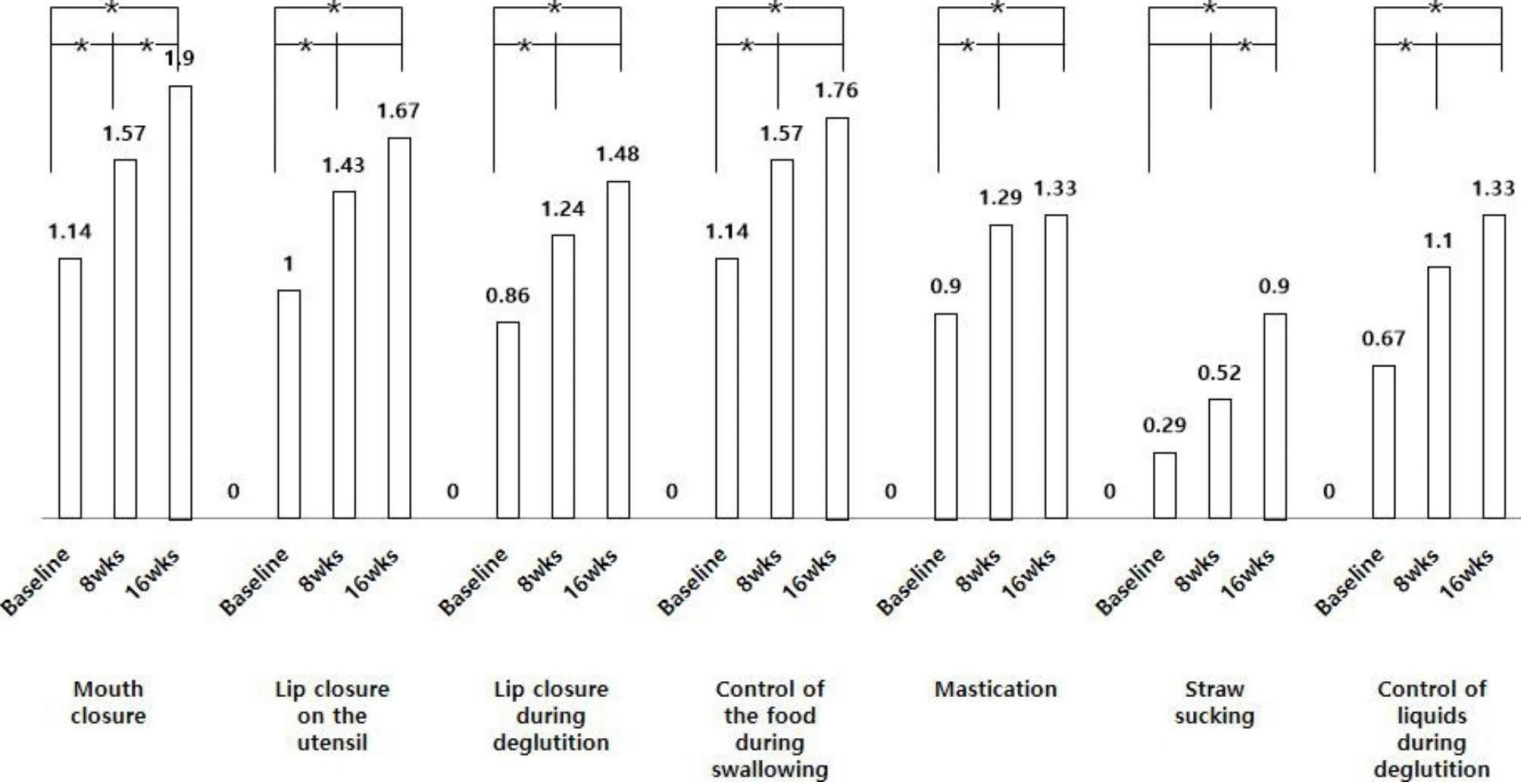
Post hoc anaylsys of OMAS items between periods of OMFT. Difference of every items between baselin and 16weeks assessment was signifcant. ^*^Post hoc analysis adjusted for *P* < 0.017

**Fig. 2:**
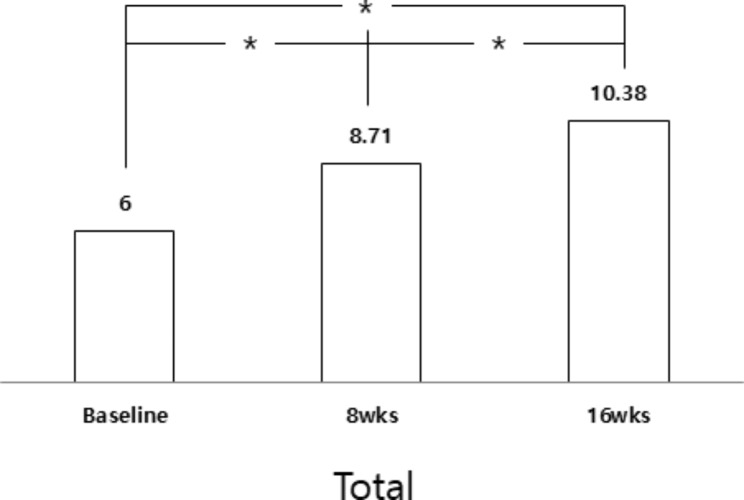
Post hoc analysis of total OMAS score between periods of OMFT. Difference between baseline, 8weeks and 16weeks assessment was significant. ^*^Post hoc analysis adjusted for *P* < 0.017

Every item of the OMAS significantly improved after 16 weeks of OMAS (Table 3). In the post hoc analysis, between baseline and 16 weeks, all items changed significantly. Between 8 and 16 weeks, mouth closure, straw suction, and total score improved significantly (Figs. 1 and 2). The treatment effect was evaluated based on the difference in the average score between baseline and 16 weeks; mouth closure was the highest (0.76), followed by straw sucking (0.62) and mastication (0.43).

**Table 3:** Effect of OMFT on oral motor and feeding skills

OMAS	Assessment stage	Mean (SD)	Mean rank	^Friedman^		Post hoc analysis		
				χ^2^	^*P*^	Assessment stage	z	^*p*^
Mouth closure	I	1.14 ± 0.57	1.40	24.13	0.000^*^	I–II	-3.00	0.003^**^
	II	1.57 ± 0.60	2.05			II–III	-2.65	0.008^**^
	III	1.90 ± 0.70	2.55			I–III	-4.00	0.000^**^
Lip closure on the utensil	I	1.00 ± 0.63	1.45	21.57	0.000^*^	I–II	-3.00	0.003^**^
	II	1.43 ± 0.81	2.10			II–III	-2.24	0.025
	III	1.67 ± 0.86	2.45			I–III	-3.74	0.000^**^
Lip closure during deglutition	I	0.86 ± 0.73	1.55	16.72	0.000^*^	I–II	-2.53	0.011^**^
	II	1.24 ± 0.70	2.10			II–III	-1.89	0.059
	III	1.48 ± 0.87	2.36			I–III	-3.13	0.002^**^
Control of the food during swallowing	I	1.14 ± 0.47	1.50	15.70	0.000^*^	I–II	-2.50	0.013^**^
	II	1.57 ± 0.60	2.12			II–III	-2.00	0.046
	III	1.76 ± 0.70	2.38			I–III	-3.15	0.002^**^
Mastication	I	0.90 ± 0.30	1.60	16.22	0.000^*^	I–II	-2.83	0.005^**^
	II	1.29 ± 0.56	2.17			II–III	-1.00	0.317
	III	1.33 ± 0.58	2.24			I–III	-3.00	0.003^**^
Straw suction	I	0.29 ± 0.46	1.60	18.89	0.000^*^	I–II	-2.24	0.025
	II	0.52 ± 0.68	1.93			II–III	-2.83	0.005^**^
	III	0.90 ± 0.77	2.48			I–III	-3.36	0.001^**^
Control of liquids during deglutition	I	0.67 ± 0.48	1.52	18.57	0.000^*^	I–II	-3.00	0.003^**^
	II	1.10 ± 0.54	2.12			II–III	-1.89	0.059
	III	1.33 ± 0.73	2.36			I–III	-3.13	0.002^**^
Total	I	6.00 ± 2.59	1.02	38.71	0.000^*^	I–II	-3.94	0.000^**^
	II	8.71 ± 3.40	2.14			II–III	-3.33	0.001^**^
	III	10.38 ± 4.56	2.83			I–III	-4.02	0.000^**^

I: baseline assessment, II: 8-week assessment, III: 16-week assessment

^*^Friedman *P* < 0.05

^**^Post hoc analysis adjusted *P* < 0.017

### Voice of parents

Here are some responses from the caregivers.

I’ve never experienced comprehensive oral motor therapy like OMFT. Especially, I didn’t know that postural control and sensory adaptation was a part of oral motor therapy (P1).

My son started breathing through his nose. And, he tried to control food in his mouth after direct oral structure facilitation by an occupational therapist (P2).

My goal was straw drinking. After I understood the developmental status of my daughter, I changed the goal of my daughter’s feeding according to her current ability (P3).

My family could eat out. Before participating in OMFT, I should carry extra food for my kids. After OMFT, My son could eat chopped pork BBQ in the restaurant (P4).

## Discussion

Feeding problems due to poor oral motor functions are very common in CP. Therefore, oral motor therapy is essential for CP with feeding problems.

Comprehensive oral motor therapy, OMFT is an effective method to enhance oral motor functions and feeding skills in CP. The total OMAS score gradually increased from baseline (6.00 ± 2.59), 8 weeks (8.71 ± 3.40) to 16 weeks (10.38 ± 4.56). In post hoc analysis, every item improved significantly after 16 weeks of OMFT. In the study of Baghbadorani et al. (2014), 12 CP participated in 8 weeks (24 sessions) of oral motor therapy including tongue exercise, lip closure exercise and chewing [15]. They assessed oral motor function before, 4 weeks and 8 weeks of treatment. After traditional oral motor therapy, all OMAS items improved. However, in post hoc analysis, only four items, including mouth closure, control of the food during swallowing, control of liquids during deglutition, and mastication, changed significantly between baseline and 8 weeks. And, only four items; control of food during swallowing, control of liquids during deglutition and mastication, and total score were significantly increased between the baseline and 4 weeks.

The effectiveness of comprehensive oral motor therapy was supported by Serel-Arslan et al.(2017) [22] and Sigan et al.(2013) [10]. Postural control, oral tactile and proprioception stimulation and other oral motor therapy techniques like chewing control improved swallowing, chewing, and drooling control [10, 22]. However, they provided only basic stimulation and postural control, and focused on specific skills like chewing is different from OMFT. Furthermore, the authors of previous studies suggested that postural control and sensory approaches should be considered together [10, 22]. And, Gisel(1994) stated that sensorimotor treatment is more effective than only providing chewing therapy in delicate oral motor function [17].

The uniqueness of OMFT is that it consists of various aspects such as postural control of the head and neck, nasal breathing, sensory adaptation, direct manual stroking on oral structures, and real food process and control. In traditional oral motor therapy, simple sensory stimulation or sensorimotor therapy is provided separately. Approaches are focused on the specific oral structures such as tongue and lips. And, postural control or sensory adaptation process was overlooked. However, in OMFT, adaptation process of postural control, sensory adaptation, and breathing control is prior to facilitate oral structures.

Difficulties exists among oral motor skills. Based on the average difference between baseline and 16 weeks of OMFT, the highest treatment effect was mouth closure (0.76) and mastication was the lowest (0.43). In the previous study [15], it is the same that the highest item was mouth closure (1.33), while the lowest item was straw suction (0.32). For example, mouth closure is simple and easy oral motor movement. However, mastication and straw suction are high-level oral function that require complex oral motor coordination, such as breathing control, swallowing timing, attention, and oral motor control. Mastication involves a systematic sensorimotor combination of bolus transfer to the molar side by the tongue, placing bolus between the tongue and cheek, safe and repetitive chewing and grinding, and moving the bolus to the back of the mouth [8, 12]. Straw drinking requires lip sealing around the straw, and the continuous sucking of liquid [17]. In Gisel’s study (1994), no improvement in straw drinking was observed after traditional oral motor therapy [17]. The treatment effect result of this study (0,61) was two times higher than that reported in a previous study (0.32) [15]. This suggests that OMFT is more effective than traditional oral motor treatment in straw drinking.

In the present study, both 8 and 16 weeks of OMFT were effective, and 16 weeks was more effective. This result is similar to the finding that 20 weeks is more effective than 10 weeks in Gisel’s study (1994), 8 weeks was more effective than 4 weeks in the study of Baghbadorani et al. (2014). Although all studies indicate that longer treatment is beneficial, each period in the three studies was different. Therefore, additional research should be performed to identify the most effective period. According to this study, at least 8 sessions of 8 weeks was needed to enhance oral motor function, however, the effective periods may depend on the development and function of children.

In prior studies [3, 15, 22], most of the participants could follow instructions or participate in feeding activities voluntarily. However, in this study, most children were in GMFCS Level IV and V. That means they couldn’t follow instruction and had limitation in voluntarily participation in feeding activity. According to the results of this study, oral motor functions and feeding skills were increased in children with perceptual limitations after OMFT. This was the reason that opportunities were provided for the first step of OMFT, sensory adaptation and postural control and breathing control. And, participants didn’t have any experience to get systematic oral motor therapy like OMFT before in South Korea context.

This study has limitations. First, the sample size was small and all children were recruited from one hospital. Therefore, generalization is not possible. Second, it was not recruited a control group. Comparison between OMFT and other traditional oral motor treatments and between groups was not possible. Third, anamnesis regarding previous oral motor therapy or related feeding therapy experience was not performed. As previous therapy experiences can influence a child’s feeding problems, pre-checking is the essential procedure. The result of this study was only verified in a small size of children with CP. Therefore, identifying the effect of OMFT on the large sample sizes with various ages and diagnoses is needed in future research.

Nevertheless, this study has several clinical indications. Our results suggest that OMFT is an effective oral motor therapy protocol to improve the oral motor function and feeding skills of CP. Comprehensive approaches are considered for providing oral motor therapy to children. And, at least 8weeks of OMFT is needed, 16 weeks of treatment is more effective. Lastly, OMFT is effective for children with CP who have audible and visible limitations.

## Conclusion

Comprehensive oral motor therapy for CP with feeding problems due to oral motor problems is essential to enhance oral motor function and feeding skills. OMFT was used to provide comprehensive oral motor therapy including postural control, sensory stimulation, breathing control and direct manual facilitation. At least 8 weeks should be needed to increase oral motor function and feeding skills. To obtain the most effective treatment results, we should understand the causes of feeding problems, and various treatments will be considered along with oral motor therapy.

## Data Availability

The data used to support the findings of this study are available from the corresponding author upon request.
